# Mesenchymal Stem Cell-Derived Extracellular Vesicles Improve the Renal Microvasculature in Metabolic Renovascular Disease in Swine

**DOI:** 10.1177/0963689718780942

**Published:** 2018-06-28

**Authors:** Alfonso Eirin, Xiang-Yang Zhu, Sreela Jonnada, Amir Lerman, Andre J. van Wijnen, Lilach O. Lerman

**Affiliations:** 1 Divisions of Nephrology and Hypertension, Mayo Clinic, Rochester, MN, USA; 2 Cardiovascular Diseases, Mayo Clinic, Rochester, MN, USA; 3 Orthopedic Surgery, Mayo Clinic, Rochester, MN, USA

**Keywords:** metabolic syndrome, renovascular disease, mesenchymal stem cells, extracellular vesicles, microcirculation

## Abstract

**Background:**

Extracellular vesicles (EVs) released from mesenchymal stem/stromal cells (MSCs) mediate their paracrine effect, but their efficacy to protect the microcirculation of the kidney is unknown. Using a novel swine model of unilateral renovascular disease (RVD) complicated by metabolic syndrome (MetS), we tested the hypothesis that EVs would attenuate renal microvascular loss.

**Methods:**

Four groups of pigs (*n* = 7 each) were studied after 16 weeks of diet-induced MetS and RVD (MetS+RVD), MetS+RVD treated 4 weeks earlier with a single intra-renal delivery of EVs harvested from autologous adipose tissue-derived MSCs, and Lean and MetS Sham controls. Stenotic-kidney renal blood flow (RBF) and glomerular filtration rate (GFR) were measured in-vivo (fast CT), whereas EV characteristics, renal microvascular architecture (micro-CT), and injury pathways were studied ex-vivo.

**Results:**

mRNA sequencing and proteomic analysis revealed that EVs are packed with several pro-angiogenic genes and proteins, such as vascular endothelial growth factor. Labeled EVs were detected in the stenotic kidney 4 weeks after injection internalized by tubular and endothelial cells. EVs restored renal expression of angiogenic factors and improved cortical microvascular and peritubular capillary density. Renal apoptosis, oxidative stress, tubular injury, and fibrosis were also attenuated in EV-treated pigs. RBF and GFR decreased in MetS+RVD compared with MetS, but normalized in MetS+RVD+EVs.

**Conclusions:**

Intra-renal delivery of MSC-derived EVs bearing pro-angiogenic properties restored the renal microcirculation and in turn hemodynamics and function in chronic experimental MetS+RVD. Our study suggests a novel therapeutic potential for MSC-derived EVs in restoring renal hemodynamics in experimental MetS+RVD.

## Introduction

Metabolic syndrome (MetS) is a cluster of cardiovascular disease-related risk factors that is frequently associated with chronic kidney disease (CKD), and increases its progression toward end-stage renal disease^1,2^. Renovascular disease (RVD) produces chronic underperfusion of the renal parenchyma, leading to progressive loss of renal mass and function^3^. Coexisting MetS and RVD are linked to poorer outcomes after revascularization^4^, underscoring the need for targeted interventions capable of preserving the post-stenotic kidney in subjects with MetS.

Mesenchymal stem/stromal cells (MSCs) are fibroblast-like multipotent adult cells present in multiple tissues with the ability to differentiate into several cell types. These cells possess unique vasculoprotective properties^5^, and their exogenous delivery has proven to be effective in restoring renal structure and function in several animal models of renal disease^6^. We have previously shown in swine RVD that intra-renal delivery of MSCs improved renal function and structure and reduced tissue injury beyond the stenotic lesion^7^. Furthermore, MSC delivery in conjuction with renal artery revascularization improves renal function and structure, and reduced oxidative stress, apoptosis, fibrosis, and microvascular remodeling in the stenotic kidney^8,9^. Importantly, with the increasing clinical translation testing the safety and efficacy of MSCs in patients, it is imperative to elucidate the mechanisms underlying their beneficial effects.

A large body of evidence indicates that an important mechanism by which MSCs confer protection is the release of extracellular vesicles (EVs), small membrane particles that express characteristics of their parental cells, can be internalized into recipient renal cells, and activate a proliferative program by delivering protein, mRNA, and micro-RNA (miR) content^10–12^. In line with this, we have recently shown that MSC-derived EVs are selectively enriched for pro-angiogenic genes and proteins, and miRNA that regulate angiogenesis^13,14^. Among them are the potent angiogenic factor vascular endothelial growth factor (VEGF), as well as activators and effectors of the Notch signaling pathway, an evolutionarily conserved signaling system that regulates vascular remodeling and arterial fate of endothelial cells. However, the implications of these observations on the renal microvasculature, and whether delivery of MSC-derived EVs preserves the structure and function of the renal microcirculation, remain to be elucidated. Therefore, using a novel swine model of MetS+RVD, we tested the hypothesis that EVs would attenuate microvascular loss in MetS+RVD.

## Materials and Methods

**Figure 1.**
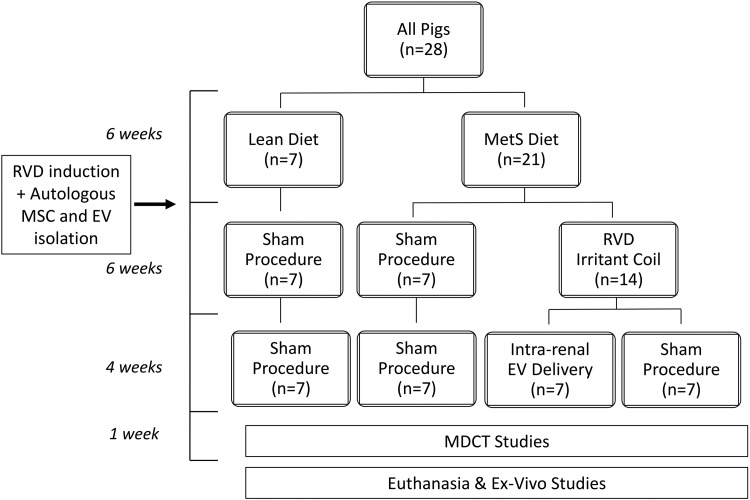
Schematic of the experimental protocol. At baseline, pigs were fed either a MetS (*n* = 21) or Lean diet (*n* = 7). Six weeks later, RVD was induced in 14 MetS pigs, whereas 7 Lean and 7 MetS pigs underwent a sham procedure. Six weeks after induction of RVD, MetS+RVD pigs received a single intra-renal infusion of either autologous MSC-derived EVs or vehicle (*n* = 7 each). Other MetS and Lean pigs underwent sham procedures (*n* = 7 each). Four weeks later, pigs were studied in-vivo and ex-vivo.

All animal procedures were approved by the Institutional Animal Care and Use Committee (approval case number: A00003694-18). Twenty-eight domestic female pigs were studied for 16 weeks (Fig. 1). At baseline, 21 pigs started a high-cholesterol/high-fructose diet (MetS)^15^ for the entire course of the study, whereas the other seven were fed regular pig chow (Lean).

Six weeks after baseline, pigs were anesthetized with 0.25 g of IM tiletamine hydrochloride/zolazepam hydrochloride (Telazol®, Zoetis, INC, Kalamazoo, MI, USA) and 0.5 g of xylazine (Xylamed, VetOne, Manufacturer is Bimeda,-MTC Animal Health, Cambridge, ON, Canada), and maintained with intravenous ketamine (0.2 mg/kg/min, [Ketaset, Distributed by Zoetis, INC, Kalamazoo, MI, USA]) and xylazine (0.03 mg/kg/min). Unilateral RVD was induced in 14 MetS pigs by placing a local-irritant coil in the main renal artery^16^, whereas 7 Lean and 7 MetS pigs underwent a sham procedure. In all animals randomized to receive EVs, fat tissue was collected at that time, and subsequently used to harvest autologous MSCs and isolate their EVs.

Six weeks after induction of RVD, the degree of stenosis in each animal was determined using renal angiography. In addition, MetS+RVD pigs received a single infusion of either autologous EVs (labeled with the red fluorescence dye PKH26, Sigma) or vehicle into the stenotic kidney over 5 min (*n* = 7 each). Two other groups of MetS and Lean pigs (*n* = 7 each) that underwent only sham procedures (angiography, saline infusion) served as controls.

Systemic blood samples were collected 4 weeks later for cholesterol fractions, isoprostanes (enzyme immunoassay kit), and plasma renin activity (PRA, GammaCoat kit; DiaSorin) levels. Fasting glucose and insulin levels were measured by standard procedures, and insulin resistance calculated by homeostasis model assessment of insulin resistance (HOMA-IR)^15^. In addition, single-kidney hemodynamics and function were determined using multi-detector computed tomography (MDCT). Arterial blood pressure was measured with an intra-arterial catheter during MDCT studies.

Pigs were euthanized with an intravenous bolus of 100 mg/kg of sodium pentobarbital (Sleepaway, Fort Dodge Inc., Fort Dodge, IA, USA) a few days after MDCT studies^17^. Kidneys were removed, dissected, and sections frozen in liquid nitrogen (and maintained at –80°C) or preserved in formalin for histology and ex-vivo studies. In addition, a lobe of kidney tissue was perfused and prepared for micro-CT studies.

### In-Vivo Studies

MDCT (Somatom Sensation-128, Siemens Medical Solution, Forchheim, Germany) scanning was performed to calculate renal volume, renal blood flow (RBF), and glomerular filtration rate (GFR), as previously shown^18–20^. Briefly, 140 consecutive scans (330 ms each) were acquired following an intra-superior vena cava bolus of iohexol (350 mg/ml over 2 seconds, [GE Healthcare, Inc. Marlborough, MA, USA]). Analyze™ (Biomedical Imaging Resource, Mayo Clinic, Rochester, MN) was used to trace cortical and medullary regions of interest, which were then used to calculate single kidney regional perfusion using MATLAB 7.10 (MathWorks). Renal volume was calculated using planimetric methods, RBF by summing cortical perfusion times cortical volume and medullary perfusion times medullary volume, and GFR from the cortical curve slope^21^.

### Ex-Vivo Studies

#### MSC and EV Isolation, Characterization, and Culture

MSCs were isolated from abdominal subcutaneous adipose tissue (5–10 g) using collagenase with standard protocol. Cells were cultured with advanced MEM medium (Gibco/Invitrogen) supplemented with 5% platelet lysate (Mayo Clinic Transfusion Medicine) in 37°/5% CO_2_^22^, and kept in cell recovery medium at –80°C for characterization. MSCs were characterized by the expression of common MSC markers (CD44, CD90, and CD105)^8,13^, and their potential to differentiate into adipocytes, chondrocytes, and osteocytes was assessed as previously described^8,23,24^.

EVs were isolated from supernatants of MSCs (10×10^6^), cultured in advanced MEM medium without supplements, and ultra-centrifuged twice, as previously described^13^. Briefly, after two initial centrifugations at 2000 *g* and 100,000 *g* (Beckman Coulter Optima L-90 K) for 1 h at 4°C, EVs were washed in serum-free medium 199 containing HEPES 25 mM, and underwent a final ultra-centrifugation. Lastly, pellets were suspended and protein content quantified (Bradford method, BioRad). Limulus testing was performed to rule out endotoxin contamination (Charles River Lab.), and EVs stored at –80°C^25^ until delivery.

Transmission electron microscopy was performed to investigate size and structure of MSC-derived EVs using digital electron microscopy (JEOL 1200 EXII, Mayo Clinic’s electron microscopy core)^26^. EVs were then characterized based on the expression of EV (CD40, ß1, CD9, and CD81), and MSC (MHC-class I and CD44) surface markers using fluorescence-activated cell sorting.

#### EV mRNA and Protein Cargo

mRNA was isolated from MSC-derived EVs using the mirVana PARIS total RNA isolation kit (Life Technologies) according to the manufacturer’s protocol. mRNA sequencing was performed at the Mayo Clinic Bioinformatic Core, as previously described^13^. Samples were sequenced on an Illumina HiSeq 2000 using TruSeq SBS kit version 3 and HCS v2.0.12 data collection software and data analyzed using the MAPRSeq v.1.2.1 system and the Bioinformatics Core standard tool, which includes alignment with TopHat 2.0.6^27,28^ and gene counts with the featureCounts software^29^. Normalized expression values for each gene were calculated as reads per kilobase per million (RPKM).

In addition, liquid chromatography mass spectrometry (LC-MS/MS) proteomic analysis was performed as previously described^30,31^. EV pellets were solubilized and lysed, and protein samples denatured. Aliquots were resolubilized in reducing sample buffer and samples electrophoresed. Gel sections were digested with trypsin^31^, and peptides extracted and transferred onto a PicoFrit column 9 (NewObjective), self-packed with Agilent Poroshell 120 S 2.7 µm EC-C18 stationary phase, using a Dionex UltiMate 3000 RSLC LC system (Thermo-Fisher Scientific). Peptides were separated and eluting peptides analyzed using a QExactive mass spectrometer (Thermo-Fisher Scientific).

#### EV Tracking

Labeled EVs were tracked and localized in frozen 5 μm sections of the stenotic kidneys by immunofluorescence staining with CD31, the distal tubular marker peanut agglutinin (PA, Vector Lab), and the proximal tubular marker *Phaseolus vulgaris* erythroagglutinin (PHA-E, Vector Lab).

#### Microvascular Density and Angiogenesis

Renal microvascular architecture was assessed using micro-CT. Renal segments were flushed with an intravascular contrast agent through a cannula ligated in a branch of each renal artery. Samples were prepared and scanned, and images analyzed as previously described^20^. Spatial density of cortical microvessels and microvascular tortuosity were calculated using Analyze™. Peritubular capillaries were counted in hemotoxylin and eosin stained slides at ×100 magnification, and the ratio of capillary number to tubules calculated^9^. Angiogenic activity was also assessed by renal VEGF immunoreactivity and immunofluorescence staining for the NOTCH family proteins Notch-1 and the endothelial Notch ligand delta-like-4 (DLL4, Abcam)^32^.

#### Renal Injury Pathways

Apoptosis was assessed in 5-μm mid-hilar cross-sections of the kidney stained with caspase-3^33^, with positive cells manually counted in 15–20 fields under fluorescence microscopy (ZEN® 2012 blue edition, Carl ZEISS). Renal endothelial cell apoptosis was assessed by double immunofluorescence staining with terminal deoxynucleotidyl-transferase dUTP nick-end-labeling (TUNEL) and the endothelial marker CD31. Renal oxidative stress was evaluated by the in-situ production of superoxide anion (dihydroethidium, DHE)^34^, and renal endothelial cell oxidative stress by double staining with nitrotyrosine and CD31. Tubular injury was assessed in sections stained with periodic acid-Schiff (PAS), as described^35^, whereas tubulo-interstitial fibrosis and glomerular score (% of sclerotic out of 100 glomeruli) were assessed in trichrome stained slides.

### Statistical Methods

Results were expressed as mean±SD. Parametric (ANOVA/Student *t*-test) and nonparametric (Wilcoxon/Kruskal–Wallis) tests were used as appropriate. All analyses were performed in JMP software package version 10.0 (SAS Institute Inc.) and significance was accepted for *p* ≤ 0.05.

## Results

The systemic characteristics in all pigs at the end of the study are summarized in Table 1. Body weight and blood pressure were comparably higher in all MetS groups compared with Lean. Pigs with RVD showed moderate, but significant stenoses (*p* > 0.05 ANOVA). Total cholesterol, high-density lipoprotein (HDL), low-density lipoprotein (LDL), and triglyceride levels were similarly elevated in all MetS groups compared with Lean. Fasting glucose levels were similar among the groups, yet fasting insulin and HOMA-IR levels were higher in MetS, indicating early pre-diabetic MetS^15^.

**Table 1.** Systemic Characteristics and Single-Kidney Function in Study Groups at 16 Weeks.

	Lean	MetS	MetS+RVD	MetS+RVD+EVs
Body weight (kg)	72.7 ± 4.6	93.8 ± 0.9*	90.7 ± 1.8*	88.9 ± 2.9*
Mean arterial pressure (mmHg)	103.8 ± 4.1	125.5 ± 4.6*	131.7 ± 7.1*	122.2 ± 2.9*
Degree of stenosis (%)	0	0	65.0 ± 8.0*	66.7 ± 3.3*
Total cholesterol (mg/dl)	81.4 ± 7.8	390.3 ± 109.5*	404.2 ± 52.2*	402.2 ± 135.3*
HDL cholesterol (mg/dl)	49.0 ± 5.5	120.3 ± 19.5*	114.2 ± 29.1*	126.7 ± 26.8*
LDL cholesterol (mg/dl)	35.1 ± 5.8	358.0 ± 124.0*	303.7 ± 81.2*	346.8 ± 82.6*
Triglycerides (mg/dl)	7.4 ± 2.1	18.3 ± 9.9*	18.8 ± 11.7*	12.9 ± 5.1*
8-isoprostane (pg/ml)	84.1 ± 10.6	139.9 ± 23.2*	259.0 ± 92.1*^†^	135.5 ± 49.7*^‡^
Fasting glucose (mg/dl)	132.5 ± 43.9	118.8 ± 25.3	114.0 ± 29.8	102.6 ± 30.5
Fasting insulin (µU/ml)	0.40 ± 0.12	0.75 ± 0.06*	0.72 ± 0.08*	0.71 ± 0.07*
HOMA-IR score	0.7 ± 0.1	1.8 ± 0.2*	1.8 ± 0.1*	1.9 ± 0.1*
Renal volume (ml)	134.6 ± 7.0	221.6 ± 7.6*	177.8 ± 8.9*^†^	228.9 ± 20.7*^‡^
RBF (ml/min)	515.8 ± 28.1	860.5 ± 89.0*	618.0 ± 30.0*^†^	861.1 ± 101.4*^‡^
GFR (ml/min)	79.2 ± 2.8	146.8 ± 7.1*	95.0 ± 5.5*^†^	134.4 ± 14.5*^‡^
Plasma renin activity (ng/ml/h)	0.16 ± 0.08	0.17 ± 0.06	0.17 ± 0.11	0.16 ± 0.11

HDL: high-density lipoprotein; LDL: low-density lipoprotein; HOMA-IR: homeostasis model assessment of insulin resistance; RBF: renal blood flow; GFR: glomerular filtration rate. *p < 0.05 vs. Lean; ^†^*p* < 0.05 vs. MetS; ^‡^*p* < 0.05 vs. MetS+RVD.

### EV Characterization

Isolated and cultured MSC expressed CD44, CD90, and CD105, and transdifferentiated into osteocytes, chondrocytes, and adipocytes, as previously shown^8,23,24^.

**Figure 2.**
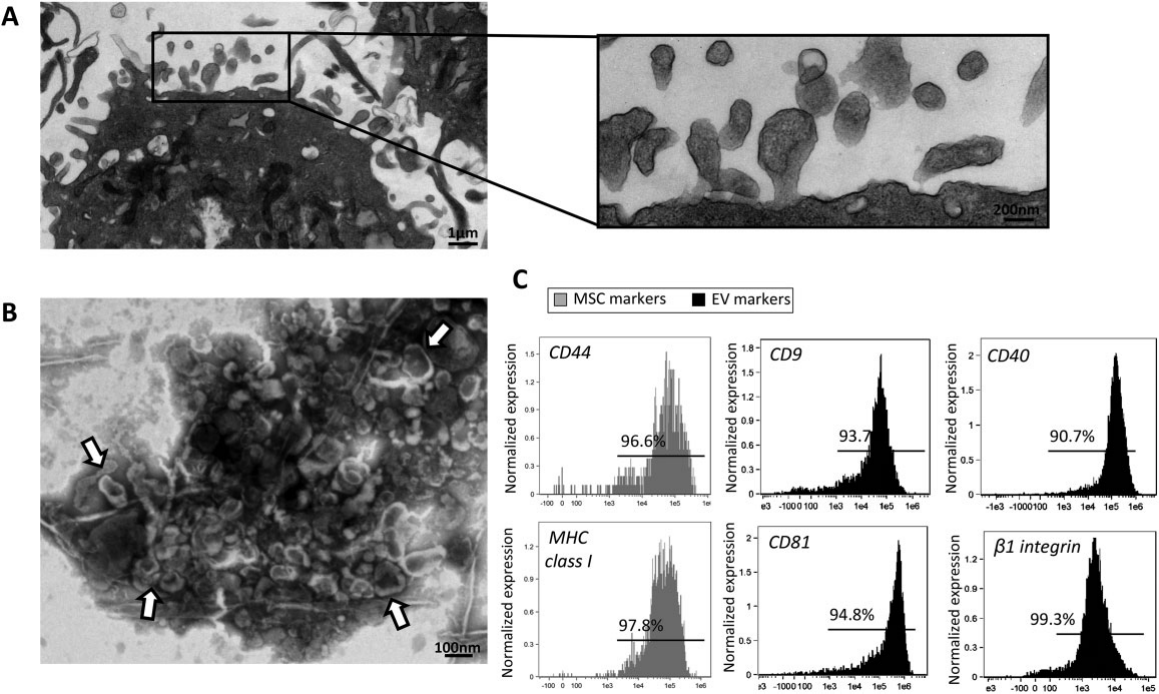
A: Transmission electron microscopy showing EVs released from MSCs. B: EVs exhibit a classic morphology on negative staining. C: Fluorescence-activated cell sorting reveals that EVs express common EV and MSC markers.

Transmission electron microscopy showed that MSCs release large amounts of EVs that exhibit the classic “cup-shape” morphology on negative staining (Fig. 2A-B), and express common EV and MSC markers (Fig. 2C).

### EV Content and Engraftment

Next-generation mRNA sequencing and proteomic analysis revealed that EVs contain several pro-angiogenic genes and proteins (Tables 2 and 3). Among them are growth factors and receptors, adhesion molecules, proteases, inhibitors and matrix proteins, transcription factors, and other factors involved in angiogenesis, suggesting important pro-angiogenic potential of MSC-derived EVs.

**Table 2.** Angiogenic genes packed in MSC-derived EVs.

Official gene symbol	Gene name	Expression (RPKM)	SD
Growth factors and receptors:			
VEGFA	Vascular Endothelial Growth Factor A	43.56	8.84
VEGFC	Vascular Endothelial Growth Factor C	14.28	2.81
FLT1	Fms Related Tyrosine Kinase 1	0.39	0.05
KDR	Kinase Insert Domain Receptor	0.01	0.001
EREG	Epiregulin	8.34	1.34
FGF2	Fibroblast Growth Factor 2	18.63	1.95
JAG1	Jagged 1	1.22	0.23
PGF	Placental Growth Factor	6.59	1.78
Adhesion molecules:			
LAMA5	Laminin Subunit Alpha 5	0.32	0.05
NRP1	Neuropilin 1	0.11	0.01
STAB1	Stabilin 1	0.15	0.02
COL4A3BP	Collagen Type IV Alpha 3 Binding Protein	1.46	0.22
IL8	C-X-C Motif Chemokine Ligand 8	0.17	0.04
Proteases, inhibitors, and matrix proteins:			
ANGPTL4	Angiopoietin Like 4	0.21	0.04
PECAM1	Platelet And Endothelial Cell Adhesion Molecule 1	0.13	0.03
PF4	Platelet Factor 4	0.82	0.25
Transcription factors:			
HAND2	Heart And Neural Crest Derivatives Expressed 2	0.06	0.01
SPHK1	Sphingosine Kinase 1	2.76	0.46
TRIM39	Tripartite Motif Containing 39	0.41	0.05
Other factors involved in angiogenesis:			
TGFBR1	Transforming Growth Factor Beta Receptor 1	3.48	0.54
CCL11	C-C Motif Chemokine Ligand 11	0.18	0.04
ENG	Endoglin	0.31	0.06
EPHB4	EPH Receptor B4	2.21	0.37
FGFR3	Fibroblast Growth Factor Receptor 3	0.18	0.03
HGF	Hepatocyte Growth Factor	7.57	1.09
IL6	Interleukin 6	23.66	4.98
PDGFA	Platelet Derived Growth Factor Subunit A	0.40	0.08
PTGS1	Prostaglandin-Endoperoxide Synthase 1	16.56	2.75
TGFB2	Transforming Growth Factor Beta 2	13.62	2.54
THBS2	Thrombospondin 2	94.93	19.26
TIMP1	TIMP Metallopeptidase Inhibitor 1	67.15	12.24
TIMP3	TIMP Metallopeptidase Inhibitor 3	148.30	26.36
WNT2	Wnt Family Member 2	1.04	0.24
WNT7B	Wnt Family Member 7B	2.21	0.35

**Table 3.** Angiogenic Proteins Packed in MSC-derived EVs.

Uniprot ID	Protein name	Expression (arbitrary units)	SD
Growth factors and receptors:			
Q8SPZ9_PIG	Vascular Endothelial Growth Factor A	11.0	2.0
F1RYW8_PIG	Epidermal Growth Factor Receptor Binding	19.0	1.6
Q7YRN5_PIG	Fibroblast Growth Factor	27.6	0.3
Adhesion molecules:			
Q5RLQ5_PIG	Neuropilin 2	16.1	2.1
F1S2I3_PIG	Collagen Type IV Alpha 3 Binding Protein	15.5	2.0
IL8_PIG	Interleukin 8	26.3	0.2
K7GLN9_PIG	Neuropilin 1	27.2	0.4
Proteases, inhibitors, and matrix proteins:			
K7GPI3_PIG	Platelet And Endothelial Cell Adhesion Molecule 1	3.7	1.5
ANGL4_PIG	Angiopoietin Like 4	11.2	2.0
ANGP1_PIG	Angiopoietin 1	21.4	1.8
K7GLK2_PIG	Vascular Cell Adhesion Molecule 1	28.9	0.2
F6PUK1_PIG	Serpin Family F Member 1	27.8	0.1
Other factors involved in angiogenesis:			
F1SRY9_PIG	Angio Associated Migratory Cell Protein	12.9	2.4
F1SJD8_PIG	Wnt Family Member 2	3.8	1.6
I3LVH1_PIG	Wnt Family Member 7B	11.0	2.0
F1RMC2_PIG	Docking Protein 2	11.3	2.1
D3K5N3_PIG	Midkine (Neurite Growth-Promoting Factor 2)	15.2	2.0
B0LDS8_PIG	Ephrin B2	11.8	2.2
I3LL31_PIG	Platelet Derived Growth Factor C	12.3	2.3
TGFB2_PIG	Transforming Growth Factor Beta 2	16.5	2.1
I3L972_PIG	Notch 3	20.6	1.7
AMC2_PIG	C-X-C Motif Chemokine Ligand 6	24.8	0.2
K7GQM6_PIG	TIMP Metallopeptidase Inhibitor 1	24.4	0.3
F1SB93_PIG	Hepatocyte Growth Factor	16.2	2.1
CCL2_PIG	C-C Motif Chemokine Ligand 2	26.1	0.2
Q8MKE5_PIG	Interleukin 6	26.8	0.1
I3LVI7_PIG	Notch 2	26.5	0.2
EGLN_PIG	Endoglin	27.1	0.3
F1SLQ6_PIG	Prostaglandin-Endoperoxide Synthase 1	30.1	0.2
PLMN_PIG	Plasminogen	27.1	0.5
Q9TTB7_PIG	TIMP Metallopeptidase Inhibitor 3	29.3	0.2

Notably, EVs also contained genes and proteins that regulate apoptosis (Tables 4 and 5) and oxidative stress (Tables 6 and 7), underscoring the potential of EVs to modulate these pathways.

**Table 4.** Antiapoptotic Genes Packed in MSC-derived EVs.

Official gene symbol	Gene name	Expression (RPKM)	SD
BNIP3	BCL2 Interacting Protein 3	0.02	0.00
CD27	CD27 Molecule	0.03	0.00
CD40LG	CD40 Ligand	0.68	0.15
DFFA	DNA Fragmentation Factor Subunit Alpha	1.96	0.30
FAS	Fas Cell Surface Death Receptor	0.01	0.00
IGF1R	Insulin Like Growth Factor 1 Receptor	1.96	0.29
MCL1	MCL1, BCL2 Family Apoptosis Regulator	18.52	3.32
NOD1	Nucleotide Binding Oligomerization Domain Containing 1	0.06	0.01
NOL3	Nucleotide Binding Oligomerization Domain Containing 3	1.43	0.20
RIPK2	Receptor Interacting Serine/Threonine Kinase 2	1.11	0.18
XIAP	X-Linked Inhibitor Of Apoptosis	2.77	0.53

**Table 5.** Angiogenic Proteins Packed in MSC-derived EVs.

Uniprot ID	Protein name	Expression (arbitrary units)	SD
C1PIG3_PIG	AKT Serine/Threonine Kinase 1	20.2	1.7
A0A0B8RTW8_PIG	BCL2 Associated Athanogene 3	13.3	2.4
F1RZR2_PIG	BCL2 Antagonist/Killer 1	26.2	0.3
A0A0B8S0B5_PIG	BCL2 Like 1	18.9	1.6
F1S402_PIG	Baculoviral IAP Repeat Containing 6	26.0	0.3
F1SJY7_PIG	BCL2 Interacting Protein 1	24.8	0.4
F1S071_PIG	BCL2 Interacting Protein 2	24.9	0.1
F1RJT5_PIG	BCL2 Interacting Protein 3 Like	12.8	2.3
F1RHS7_PIG	DNA Fragmentation Factor Subunit Alpha	11.9	2.2
TNR6_PIG	Fas Cell Surface Death Receptor	16.5	2.1
F1SRY1_PIG	Insulin Like Growth Factor 1 Receptor	26.3	0.2
K9IWB2_PIG	MCL1, BCL2 Family Apoptosis Regulator	18.6	1.6
K7GT21_PIG	X-Linked Inhibitor Of Apoptosis	12.2	2.2

**Table 6.** Antioxidant genes packed in MSC-derived EVs.

Official gene symbol	Gene name	Expression (RPKM)	SD
Glutathione Peroxidases:			
GPX1	Glutathione Peroxidase 1	24.64	4.92
GPX2	Glutathione Peroxidase 2	0.01	0.00
GPX4	Glutathione Peroxidase 4	25.76	4.87
GPX6	Glutathione Peroxidase 6	0.004	0.00
GPX8	Glutathione Peroxidase 8	10.27	1.65
GSTZ1	Glutathione S-Transferase Zeta 1	0.03	0.00
Superoxide Dismutases:			
SOD1	Superoxide Dismutase 1	2.57	0.42
SOD2	Superoxide Dismutase 2	13.83	2.04
SOD3	Superoxide Dismutase 3	3.02	0.66
Peroxiredoxins:			
PRDX1	Peroxiredoxin 1	29.96	4.44
PRDX5	Peroxiredoxin 5	0.01	0.00
PRDX6	Peroxiredoxin 6	0.39	0.06
Other Peroxidases:			
CSDE1	Cold Shock Domain Containing E1	24.51	3.19
CYGB	Cytoglobin	7.78	1.72
DUOX1	Dual Oxidase 1	0.001	0.00
EPX	Eosinophil Peroxidase	0.01	0.00
PTGS1	Prostaglandin-Endoperoxide Synthase 1	16.56	2.75
PTGS2	Prostaglandin-Endoperoxide Synthase 2	0.75	0.17
LPO	Lactoperoxidase	0.01	0.00
TPO	Thyroid Peroxidase	0.005	0.00
Other Antioxidants:			
GSR	Glutathione-Disulfide Reductase	2.32	0.38

**Table 7.** Antioxidant proteins packed in MSC-derived EVs.

Uniprot ID	Protein name	Expression (arbitrary units)	SD
Glutathione Peroxidases:			
O77732_PIG	Glutathione Peroxidase 1	29.67	0.27
GPX4_PIG	Glutathione Peroxidase 4	29.52	0.24
J9JIK0_PIG	Glutathione Peroxidase 6	12.36	2.26
I3L856_PIG	Glutathione Peroxidase 7	29.29	0.27
K7GN85_PIG	Glutathione S-Transferase Zeta 1	26.95	0.26
Superoxide Dismutases:			
Q95ME5_PIG	Superoxide Dismutase 1	29.65	0.37
Q95JF1_PIG	Superoxide Dismutase 2	30.91	0.23
I3LUD1_PIG	Superoxide Dismutase 3	24.96	0.27
Peroxiredoxins:			
F1S3U9_PIG	Peroxiredoxin 1	32.19	0.23
F1SDX9_PIG	Peroxiredoxin 2	31.36	0.29
F1S418_PIG	Peroxiredoxin 3	31.62	0.22
A0A0B8RTC3_PIG	Peroxiredoxin 4	31.31	0.19
Q9GLW8_PIG	Peroxiredoxin 5	27.78	0.30
PRDX6_PIG	Peroxiredoxin 6	32.78	0.29
Other Peroxidases:			
CATA_PIG	Catalase	31.35	0.28
F1SBS1_PIG	Cold Shock Domain Containing E1	27.73	0.40
F1RWP3_PIG	Cytoglobin	16.66	2.18
Q2EN77_PIG	Microsomal Glutathione S-Transferase 3	26.78	0.30
F1SLQ6_PIG	Prostaglandin-Endoperoxide Synthase 1	30.15	0.20
F1S9J3_PIG	Peroxidasin	30.61	0.27
Other Antioxidants:			
ALBU_PIG	Albumin	34.64	0.19
APOE_PIG	Apolipoprotein E	25.75	0.50
F1RX66_PIG	Glutathione-Disulfide Reductase	28.54	0.30
F1SG38_PIG	Thioredoxin Reductase 1	28.01	0.28
F1RHN4_PIG	Thioredoxin Reductase 2	21.66	1.79

**Figure 3.**
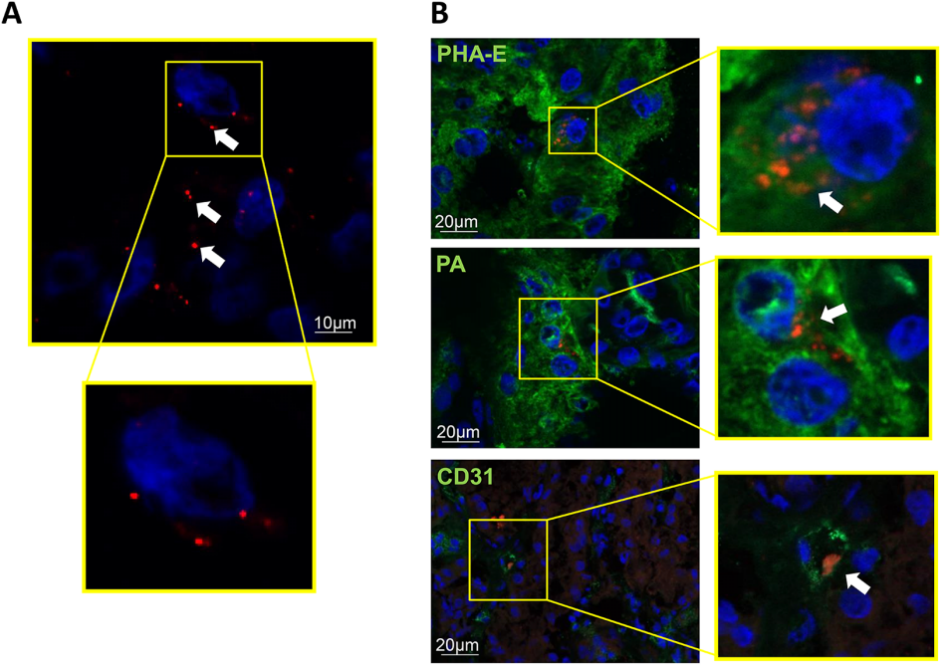
A: EV clusters were detected in the swine kidney 4 weeks after intra-renal delivery (arrows). B: Immunofluorescence co-staining with *Phaseolus vulgaris* erythroagglutinin (PHA-E), peanut agglutinin (PA), and CD31, shows EV engraftment in proximal and distal tubules, and endothelial cells, respectively.

MSC-derived EVs were detected in the post-stenotic swine kidney 4 weeks after intra-renal administration (Fig. 3A), co-localizing with proximal (PHA-E positive) and distal (PA positive) tubular cells, and endothelial cells (CD31) (Fig. 3B).

### EVs Improved the Renal Microcirculation

**Figure 4.**
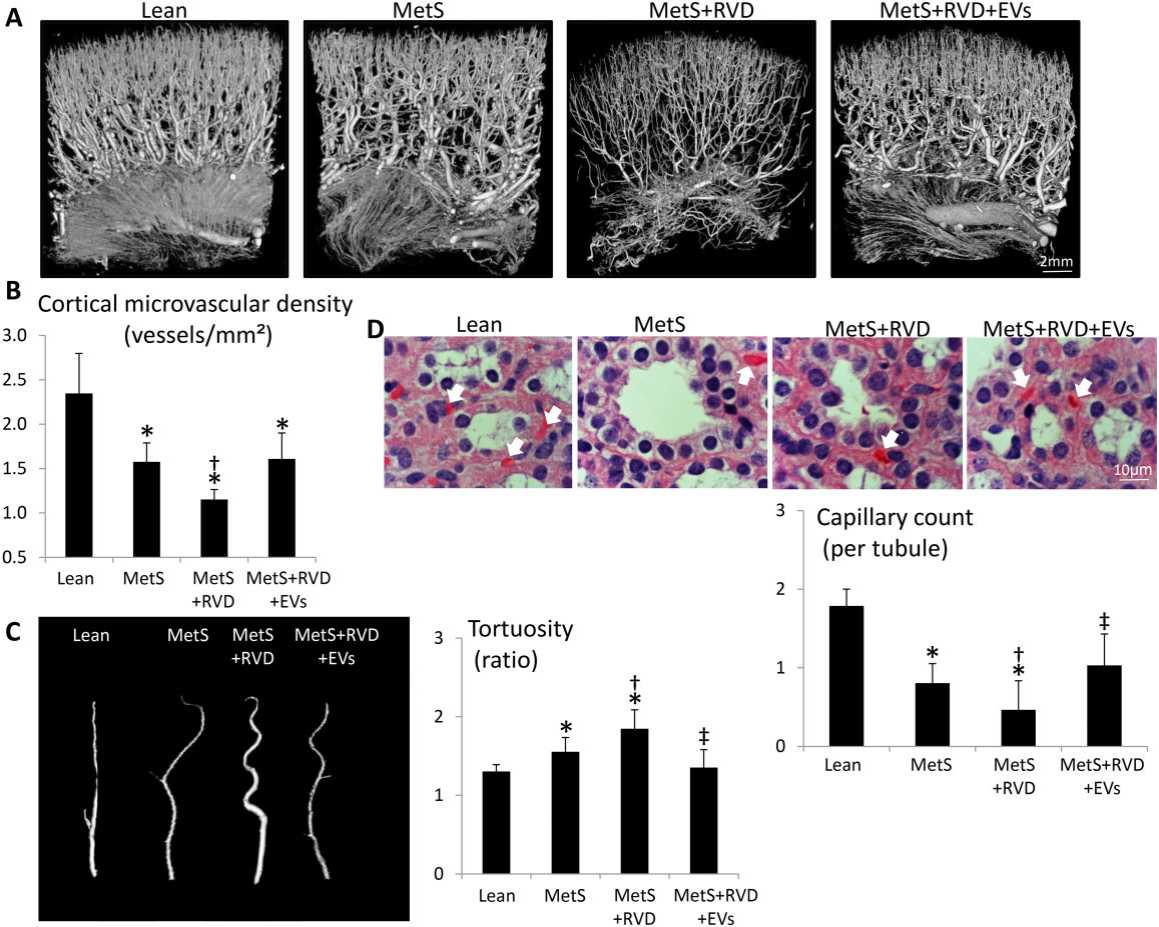
*EVs improve microvascular architecture in MetS+RVD.* A: Representative 3D micro-computed tomography images of the pig kidney showing improved microvascular architecture in EV-treated pigs. B: Quantification of spatial density of renal cortical microvessels (left) and microvascular tortuosity (right). D: Representative renal hemotoxylin and eosin (H&E) staining and quantification of peritubular capillary density. *p < 0.05 vs. Lean; ^†^*p* < 0.05 vs. MetS; ^‡^*p* < 0.05 vs. MetS+RVD.

**Figure 5.**
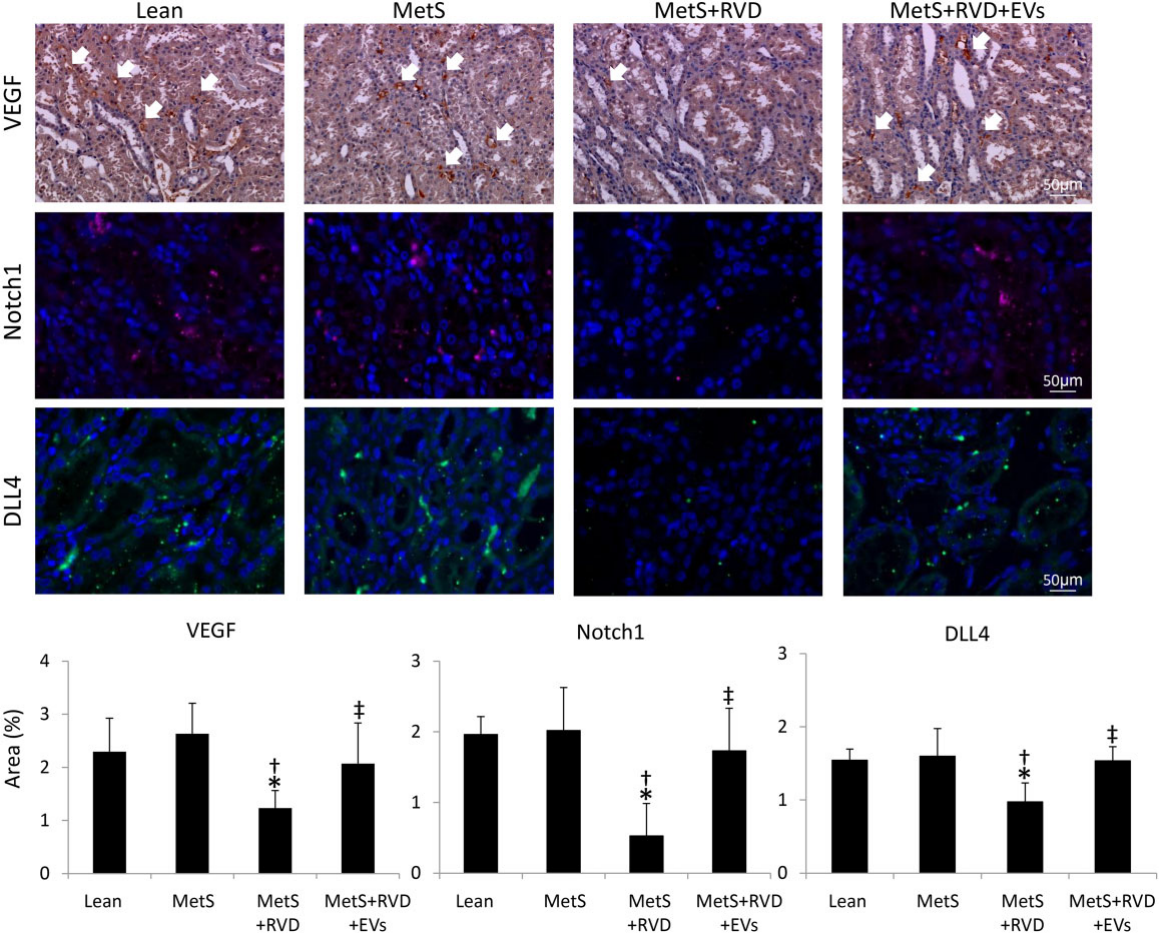
*EVs improve angiogenic signaling in MetS+RVD.* Representative stenotic kidney staining (×40) for the pro-angiogenic factors VEGF, Notch-1, and Notch ligand delta-like-4 (DLL4), and their quantification. *p < 0.05 vs. Lean; ^†^*p* < 0.05 vs. MetS; ^‡^*p* < 0.05 vs. MetS+RVD.

Spatial density of cortical microvessels was measurably diminished in MetS and further reduced in MetS+RVD, but improved (although not fully normalized) in MetS+RVD+EVs (Fig. 4A-B). Microvascular tortuosity (indicating immaturity) was higher in MetS compared with normal and further increased in MetS+RVD, but normalized in EV-treated pigs (Fig. 4C). Peritubular capillary density decreased in MetS compared with Lean, decreased further in MetS+RVD, but improved in EV-treated pigs (Fig. 4D). Immunoreactivity of the pro-angiogenic factors VEGF, Notch-1, and DLL4 was downregulated in MetS+RVD, but EVs restored their expression (Fig. 5).

### EVs Ameliorated Renal Injury

**Figure 6.**
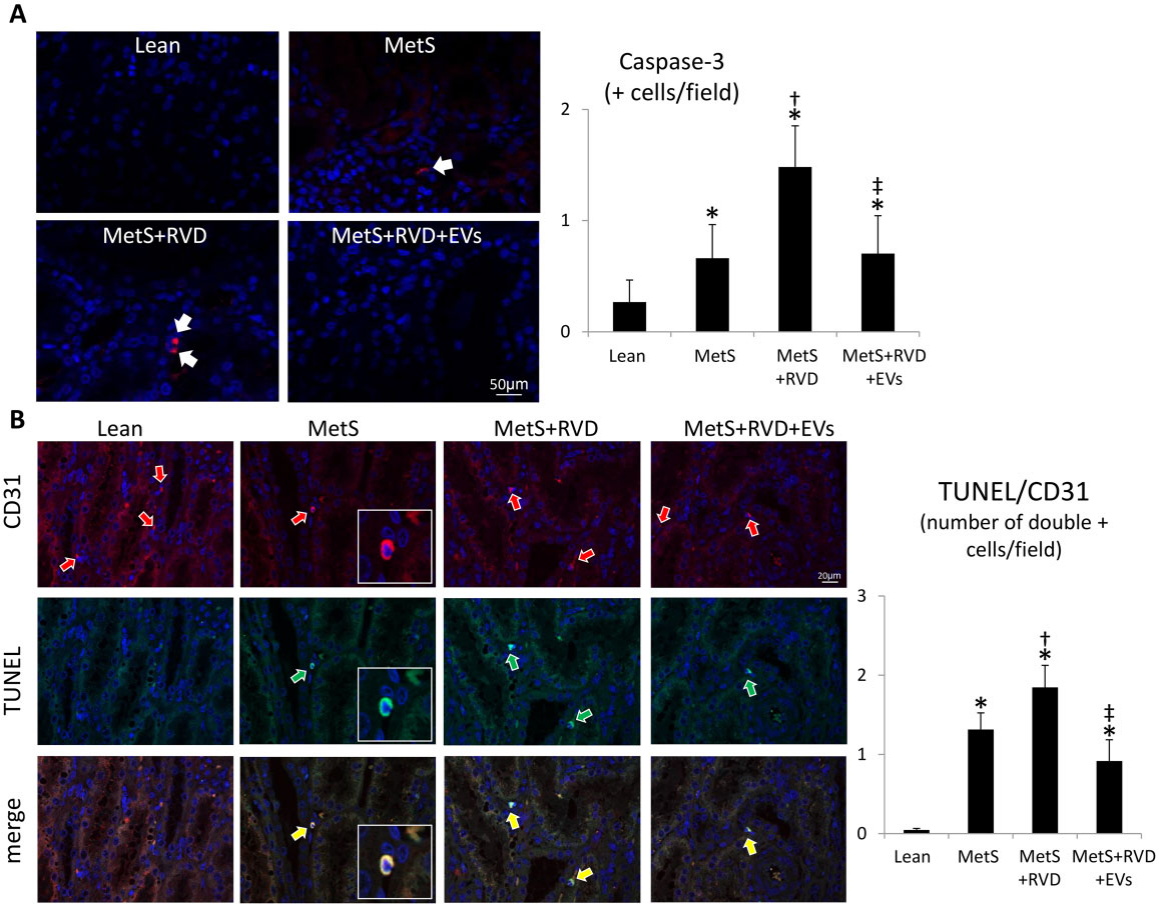
*EVs decreased endothelial cell apoptosis in MetS+RVD.* A: Fluorescent renal staining (40×) and quantification of caspase-3. B: Double renal fluorescence staining with terminal deoxynucleotidyl transferase-mediated dUTP nick end labeling (TUNEL, green arrows) and CD31 (green arrows). Yellow arrows indicated double + cells. *p < 0.05 vs. Lean; ^†^*p* < 0.05 vs. MetS; ^‡^*p* < 0.05 vs. MetS+RVD.

**Figure 7.**
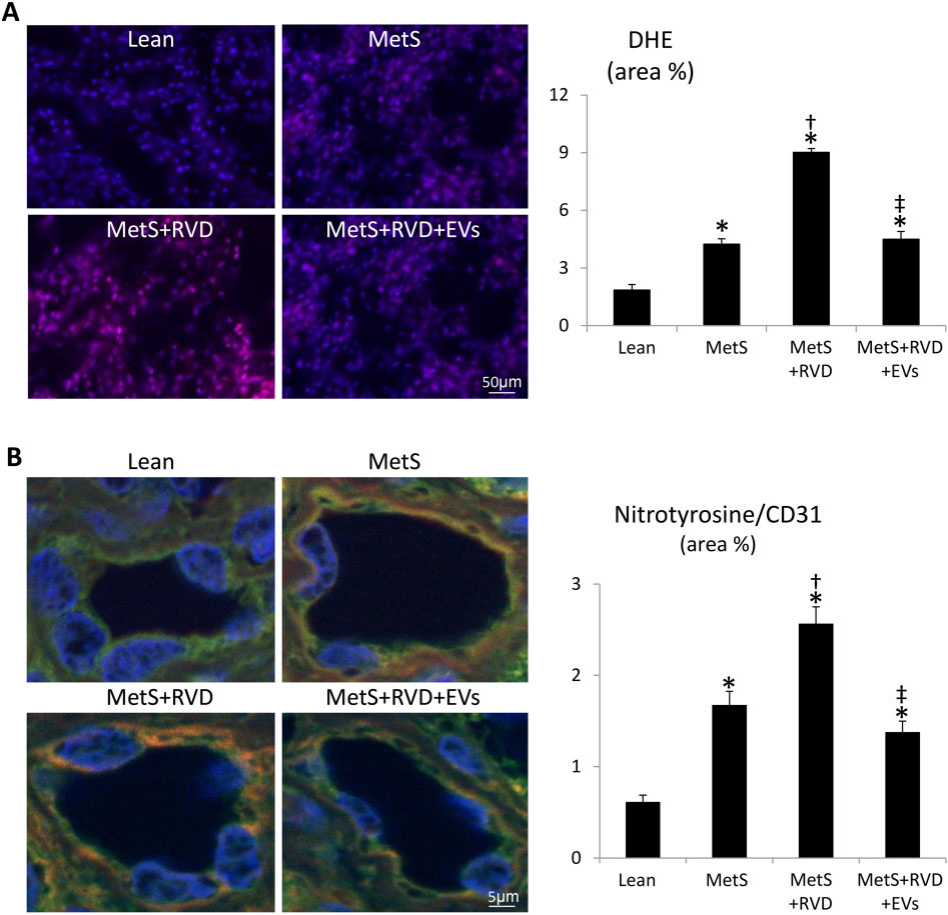
*EVs decreased vascular oxidative stress in MetS+RVD.* A: Fluorescent renal staining (40×) and quantification of dihydroethidium (DHE). Double renal fluorescence staining with nitrotyrosine (red arrows) and CD31 (green arrows) shows endothelial-cell-specific oxidative stress. Yellow arrows indicate double + cells. *p < 0.05 vs. Lean; ^†^*p* < 0.05 vs. MetS; ^‡^*p* < 0.05 vs. MetS+RVD.

**Figure 8.**
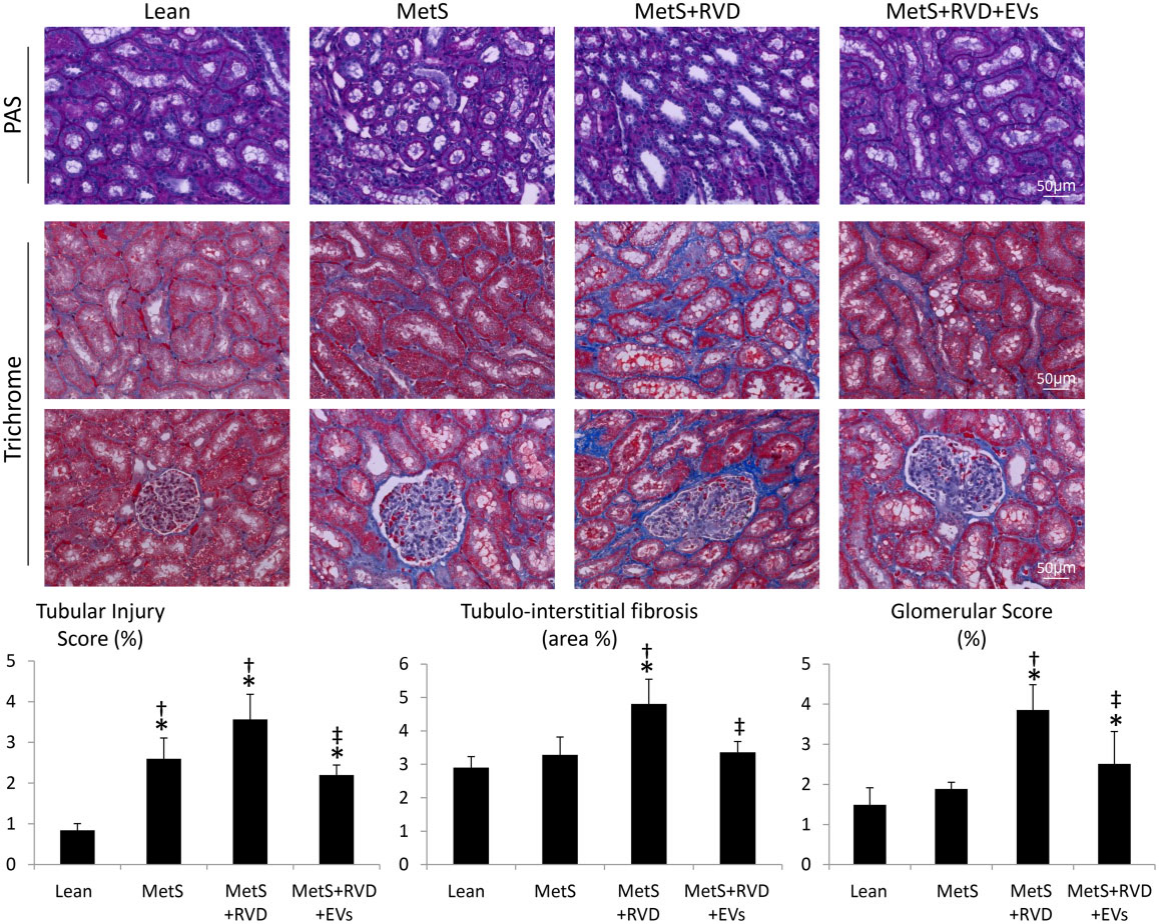
Representative kidney periodic acid-Schiff (PAS) and trichrome staining (40×), and quantifications of tubular injury, tubulo-interstitial fibrosis, and glomerular score. *p < 0.05 vs. Lean; ^†^*p* < 0.05 vs. MetS; ^‡^*p* < 0.05 vs. MetS+RVD.

The number of caspase-3+ apoptotic cells increased in MetS kidneys, further increased in MetS+RVD, but decreased in MetS+RVD+EVs (Fig. 6A), as did the number of TUNEL+/CD31+ cells (Fig. 6B), suggesting endothelial cell apoptosis. Renal production of superoxide anion (DHE) was elevated in all MetS compared with Lean, increased further in MetS+RVD, but decreased to levels comparable to MetS in MetS+RVD+EVs (Fig. 7A), as did levels of circulating isoprostanes (Table 1). Double immunoreactivity of nitrotyrosine and CD31 increased in MetS and MetS+RVD, but slightly decreased in EV-treated pigs (Fig. 7B), indicating vascular oxidative stress. Tubular injury score increased in MetS+RVD compared with Lean and MetS, but decreased in MetS+RVD+EVs, as did tubulo-interstitial fibrosis and glomerular score (Fig. 8).

### MSC-derived EVs Restored Renal Function

Single-kidney volume, RBF, and GFR were higher in all MetS compared with Lean, decreased in MetS+RVD, but increased to MetS levels in MetS+RVD+EVs (Table 1). Yet, PRA levels did not differ among the groups.

## Discussion

This study demonstrates that MSC-derived EVs preserve the microcirculation of the post-stenotic kidney in coexisting experimental MetS and RVD. A single intra-renal delivery of autologous EVs restored intra-renal expression of angiogenic factors, reduced microvascular remodeling and loss in the stenotic RVD kidney, and in turn stenotic-kidney tissue injury. Importantly, the protective effects of EVs might be attributed partly to their cargo of pro-angiogenic genes and proteins, and their capacity to improve microvascular disease, an important determinant of renal function beyond a stenotic lesion. Therefore, our observations may shed light into the mechanisms underlying the vasculo-reparative properties of MSCs, and suggest a novel cell-free refinement of MSC therapy to treat MetS+RVD.

The prevalence of MetS approaches 50% in patients presenting with symptomatic RVD, and their coexistence aggravates renal functional outcomes after revascularization^4^. MetS directly impacts on the vascular system, amplifying vessel thickness and stiffness^36^, and favoring development of atherosclerosis and thrombosis^37^. Previous studies in swine RVD have demonstrated that microvascular remodeling, damage, or loss play a critical role in the progression of renal injury. Impaired microvascular structure or function leads to tubular injury, matrix accumulation, interstitial fibrosis, and renal dysfunction^38^. Importantly, superimposition of MetS aggravates renal microvascular damage in swine RVD^39^ and blunt renal recovery in patients with RVD^4^, underscoring the need for effective therapeutic strategies to preserve the renal microcirculation.

Stem cells have shown a tremendous potential to attenuate both acute and chronic renal microvascular injury. We have previously shown in chronic swine RVD that intra-renal delivery of autologous adipose tissue-derived MSCs improves renal function^7,8^. These effects may be mediated by the release of EVs, which shuffle genes and proteins that mediate the paracrine activity of MSCs. Importantly, the cargo of MSC-derived EVs appears to be selected to activate endogenous repair mechanisms in recipient cells. We have previously shown that EVs are preferentially enriched for genes encoding transcription factors that modulate pro-angiogenic pathways, whereas genes encoding for mitochondrial, calcium signaling, and cytoskeletal proteins were selectively excluded^13^. Similarly, proteins enriched in MSC-derived EVs are linked to angiogenesis, whereas those depleted are primarily involved in nucleotide binding and RNA splicing^14^. More recently, we found that interactions among the mRNAs and microRNAs enriched in MSC-derived EVs regulate transcription factor activity in EVs and recipient cells^40^. These observations suggest that EVs have a selectively enriched cargo with a specific biological signature that promotes angiogenesis and facilitates tissue repair.

The important role of EVs as paracrine mediators of MSCs is underscored by experimental studies showing that MSCs and EVs exhibit a comparable and potentially additive effect on reducing renal injury and dysfunction^41^, and might in fact confer additional renoprotective effects. Indeed, delivery of combined MSCs and MSC-derived EVs is superior to either one alone in improving renal function^42^. In agreement with this, we have recently found that MSC-derived EVs attenuate renal inflammation and improve function in coexisting MetS and RVD^43^, mimicking the effects of MSCs in non-atherosclerotic and atherosclerotic swine RAS^7,8,24^. Likewise, a recent pilot study demonstrated that systemic administration of umbilical cord MSC-derived EVs improves kidney function in patients with CKD^44^. However, whether improvement in renal function by EV delivery is associated with preservation of the renal microvascular architecture in RVD remains unknown.

The current study used an innovative animal model integrating both MetS^15^ and RVD^16^, recapitulating features of human disease. We found that a single intra-renal delivery of adipose-tissue MSC-derived EVs provides a means to preserve microvascular architecture and recover the function of the MetS+RVD kidney. For example, the spatial density of cortical microvessels, major determinants of GFR in the stenotic kidney^34^, decreased in MetS+RVD, but slightly improved in MetS+RVD+EVs. Furthermore, intra-renal delivery of EVs improved vascular maturity, reflected in decreased vessel tortuosity^45^, and improved the number of peritubular capillaries, which maintain tubular integrity^46^.

The angiogenic potency of MSC-derived EVs, which carry protein-encoding mRNAs that stimulate vascular development (e.g. VEGF-A, VEGF-C, VEGF receptors, etc.) and proteins (e.g. VEGF, Angiopoietin Like 4, Hepatocyte Growth Factor, etc.), might have contributed to the improved renal microvasculature, as renal expression of the pro-angiogenic proteins VEGF, Notch-1, and DLL4 improved in MetS+RVD+EVs. Indeed, VEGF-induced gene expression of Notch1 and DLL4 in human arterial endothelial cells triggers arteriogenesis and angiogenesis, establishing a functional linkage between these two angiogenic signaling pathways^47^. Speculatively, the uptake of EVs carrying a pro-angiogenic cargo by CD31+ cells might have contributed to improved renal angiogenesis and microvasculature.

Notably, restoration of renal angiogenesis and microvascular architecture in EV-treated pigs was associated with decreased microvascular oxidative stress and apoptosis. We have previously shown that renal microvascular remodeling and loss in the stenotic kidney are associated with increased oxidative stress, and that antioxidant intervention improves RBF and decreases renal fibrosis^48^. Likewise, acute and chronic treatments with compounds that prevent mitochondrial-dependent apoptosis restore microvascular architecture in RVD, suggesting that apoptosis may also contribute to loss of vascular cells^33,35^. Consistent with these observations, we found that renal superoxide anion and circulating isoprostanes increased in MetS+RVD, but decreased in EV-treated pigs, whereas the number of caspase-3+ apoptotic cells decreased in MetS+RVD+EV kidneys, extending previous in-vitro studies employing EVs^25^. In addition, we used nitrotyrosine staining, a footmark of peroxynitrate-mediated protein damage, as an index for increased abundance of superoxide anion, and thus oxidative stress^49^. Indeed, renal endothelial cell apoptosis and oxidative stress increased in MetS+RVD, but decreased in EV-treated pigs. Notably, restoration of the renal microvasculature might have partly contributed to attenuate oxidative stress and apoptosis by preserving delivery of oxygen. Nevertheless, this might have been a direct antioxidant or anti-apoptotic effect of EVs, which carry genes that modulate apoptosis and oxidative stress, and incorporate into proximal and distal tubular cells and endothelial cells. For example, EVs are packed with numerous antiapoptotic genes, such as several BCL2 family apoptosis regulators and the X-linked inhibitor of apoptosis. Likewise, EVs contained several genes and proteins with important antioxidant properties, including glutathione peroxidases, superoxide dismutases, and peroxiredoxins. Overall, EV-induced microvascular proliferation likely contributed to improve RBF and renal function (GFR and serum creatinine levels), and attenuate tubular injury and fibrosis, underscoring important reno-protective properties of this intervention.

MSC-derived EVs may offer several advantages over their parent cells in repairing the post-stenotic kidney. Being acellular, EVs cannot proliferate in the recipient tissue and are thus exempted from adverse effects associated with delivery of live replicating MSCs, such as maldifferentiation or malignant transformation^50^. Although renal retention rate is similar between MSCs and their daughter EVs, their small size might allow EV penetration into deeper regions of the kidney upon injection into the renal artery, extending their reparative effects. In addition, EVs are more stable than MSCs and for practical aspects can be cryopreserved and stored for a long time, allowing their use as “off the shelf” products. Taken together, these advantages suggest that delivery of MSC-derived EVs is a useful regenerative strategy to improve the damaged kidney. On the other hand, it is possible that the ability of MSCs to survive, proliferate, and release EVs after engraftment would prolong the beneficial effects of cell-based therapy, but this postulation remains to be tested.

Our study has some limitations including the use of young animals and the short duration of MetS and RVD. Nevertheless, our MetS animals developed obesity, hypertension, hyperlipidemia, and insulin resistance, which impart subtle changes on kidney structure and function, such as hyperfiltration, microvascular loss, apoptosis, oxidative stress, and tubular injury. Moreover, as observed in human subjects^4^, superimposition of RVD markedly aggravated MetS-induced renal injury, contributing to post-stenotic dysfunction and fibrosis. We have previously shown that retention of EVs in the stenotic kidney peaked at 2 days (∼9% of injected amount) and decreased thereafter, remaining at 2% by 4 weeks after injection^43^. While EVs were clearly detected, co-localizing with proximal and distal tubular cells, and endothelial cells, the mechanisms regulating EV engraftment remain to be determined and evaluated in future studies.

In summary, our study suggests a novel therapeutic role for MSC-derived EVs in promoting angiogenesis and vascular repair, and thereby improving renal function in chronic experimental MetS+RVD. Intra-renal administration of EVs normalized renal expression of pro-angiogenic factors, improved microvascular architecture, and decreased tissue injury in the post-stenotic kidney. These observations therefore reveal that EVs are endowed with pro-angiogenic potential to repair the damaged kidney in coexisting MetS+RVD. Further studies are needed to examine how these beneficial effects compare with MSCs, and whether they persist in individuals suffering from this prevalent disease.
